# Isomerism of the right atrial appendages with bilateral eparterial bronchi: clinical challenges and surgical management guided by 3D cardiac computed tomography

**DOI:** 10.1007/s12055-025-02022-6

**Published:** 2025-08-22

**Authors:** Jainam Shah, Sachin Pathuri, Randy Richardson

**Affiliations:** 1 Albert Einstein College of Medicine, 1300 Morris Park Ave, Bronx, NY 10461 USA; 2 Creighton University School of Medicine, 3100 N Central Ave, Phoenix, AZ 85012 USA

**Keywords:** Isomerism of the right atrial appendages, Asplenia, Discordant ventriculo-arterial connections, Right-handed ventricular topology, Bilateral eparterial bronchi

## Abstract

Isomerism of the right atrial appendages is a rare congenital disorder marked by abnormal lateralization of the thoracoabdominal organs, often associated with complex cardiac malformations and asplenia. We report a neonate with discordant ventriculo-arterial connections, an anterior and right-sided aorta, total anomalous pulmonary venous return, and bilateral eparterial bronchi, identified using cardiac computed tomography with three-dimensional (3D) reconstruction. The patient underwent staged surgical interventions including a Blalock-Taussig shunt and Glenn procedure. Postoperative complications included gastroesophageal reflux and respiratory distress requiring multidisciplinary care. While not intended to guide generalized management, this case illustrates the utility of multimodality imaging and surgical planning in addressing the complex anatomy associated with isomerism of the right atrial appendages.

## Introduction

Isomerism of the atrial appendages is a rare congenital disorder occurring in approximately 1 in 10,000 live births [1]. It represents a failure of normal left–right patterning, producing bilateral structures characteristic of either right or left atrial morphology. Isomerism of the right atrial appendages is characterized by bilateral right atrial appendages, asplenia, bilateral tri-lobed lungs, and frequently, complex congenital cardiac defects [1, 2]. This contrasts with isomerism of the left atrial appendages, which is associated with polysplenia and bilateral bi-lobed lungs. With modern multimodality imaging, including echocardiography and cardiac computed tomography, almost all patients can be definitively assigned to right or left atrial isomerism, making use of older terms such as “heterotaxy syndrome” and “situs ambiguous” clinically obsolete [3, 4]. Although “heterotaxy syndrome” has historically been used and remains commonly cited in the pediatric cardiology and surgical literature [3, 4], recent proposals from cardiac morphologists advocate for classification strictly by isomerism of the atrial appendages due to advances in multimodality imaging [2]. Importantly, Hagen et al. showed that identification of disease-related genomic copy number variants is only interpretable once patients are segregated by atrial appendage isomerism [1]. In our patient, the organ arrangement was entirely consistent with isomerism of the right atrial appendages, with no discordant findings. We present this case to illustrate the role of advanced imaging and multidisciplinary care in managing this complex entity.

## Case report

A male neonate was delivered vaginally at 39 weeks and admitted for evaluation of a complex congenital heart defect. Prenatal ultrasound had previously identified transposition of the great vessels and mild pulmonic stenosis. On day-of-life 4, the patient’s oxygen saturation fell dramatically, requiring prostaglandin to maintain ductal patency and restore saturation to normal levels. The patient was then transferred to the Pediatric Cardiothoracic Intensive Care Unit for continued care and examination.

**Fig. 1:**
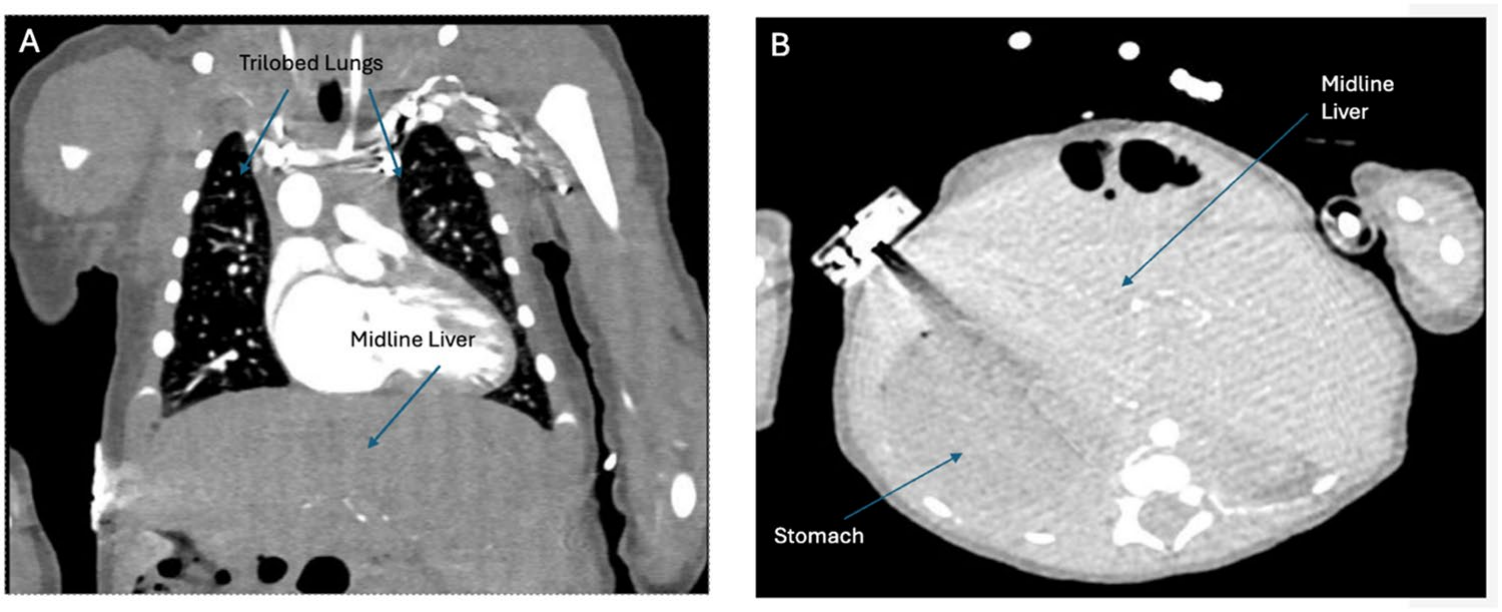
(**A**) Computed tomography angiography of the chest showing midline liver and tri-lobed right lungs bilaterally. (**B**) Transverse computed tomography scan showing midline liver and abnormal stomach placement

**Fig. 2:**
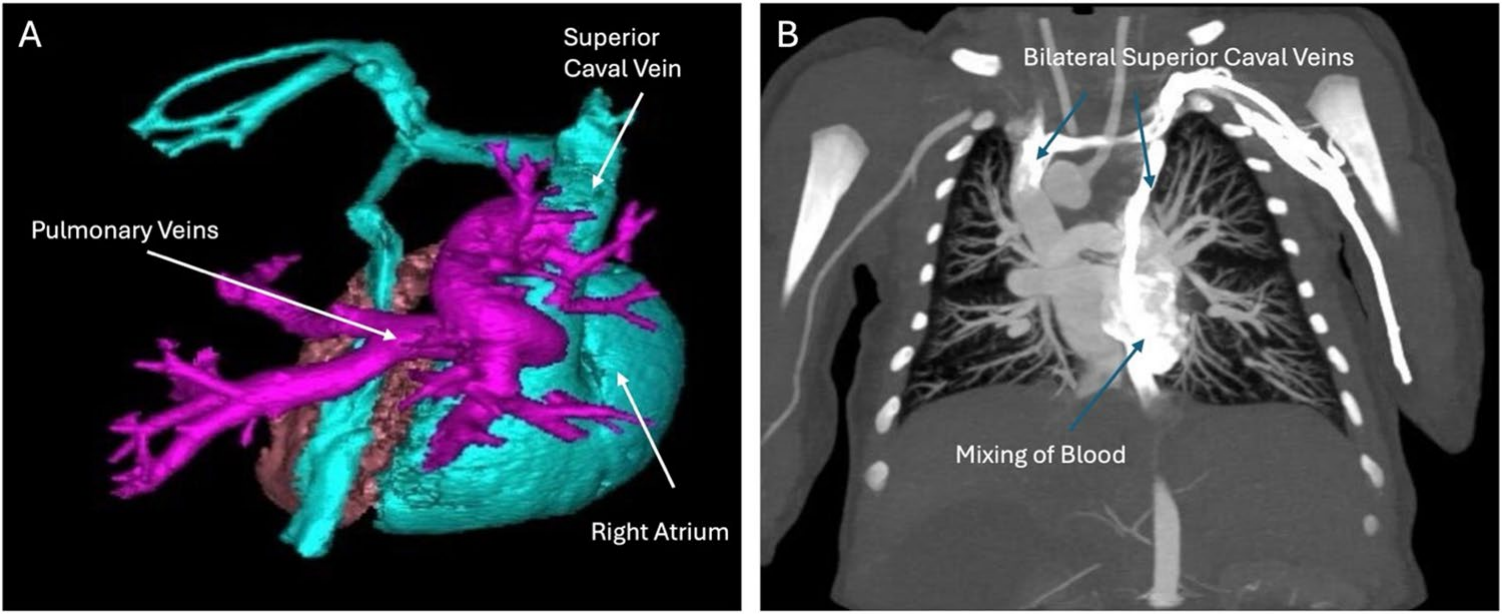
(**A**) Three-dimensional reconstruction showing total anomalous pulmonary venous return, with pulmonary veins emptying into the superior caval vein and right atrium. (**B**) Computed tomography angiography showing bilateral superior caval veins connecting to the atria, along with blood mixing due to lack of separation between the left and right ventricles

**Fig. 3:**
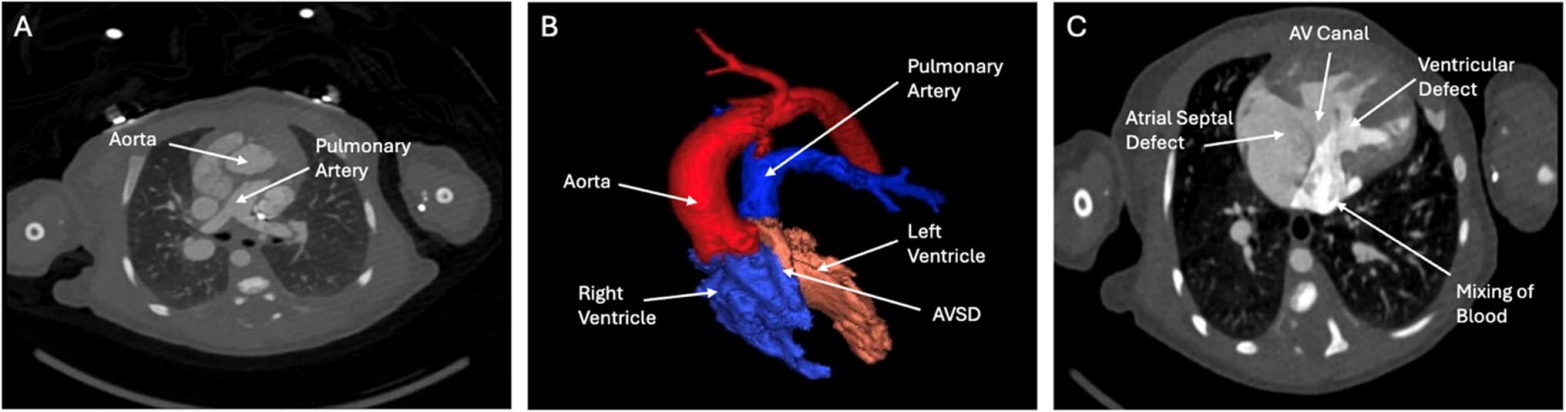
(**A**) Transverse computed tomography angiography showing discordant ventriculo-arterial connections with an anterior and right-sided aorta. (**B**) Three-dimensional reconstruction of the heart showing discordant ventriculo-arterial connections with an anterior aorta arising from the right ventricle and a pulmonary artery arising from the left ventricle, consistent with isomerism of the right atrial appendages. (**C**) Transverse computed tomography angiography showing common atrioventricular canal and atrioventricular septal defect with absence of ventricular septation

Cardiac computed tomography angiography with three-dimensional (3D) reconstruction showed isomerism of the right atrial appendages, with asplenia, midline liver, and bilateral tri-lobed lungs (Fig. 1). All pulmonary veins drained anomalously to the superior caval vein, consistent with total anomalous pulmonary venous return (Fig. 2). The heart exhibited discordant ventriculo-arterial connections with an anterior and right-sided aorta (Fig. 3), along with atrioventricular septal defect. Right-handed ventricular topology was also noted. Lastly, bilateral eparterial bronchi were observed, as both the left and right bronchi arose superior to the pulmonary artery.

At 12 days, the patient underwent surgery for the placement of a 3.5-mm Blalock-Taussig shunt, which connected the innominate artery to the left pulmonary artery to improve pulmonary blood flow. Severe gastroesophageal reflux during the postoperative period necessitated feeding through a gastronomy tube. Prophylactic amoxicillin was prescribed for asplenia. Subsequently, the patient underwent a Ladd procedure to correct intestinal malrotation and was placed on hypoallergenic infant formula at 24 kcal/oz. Following an episode of respiratory distress at 4 months of age, the patient was recommended for Glenn procedure. At 6 months of age, during the Glenn procedure, the patient’s right and left superior caval veins were connected to the right and left pulmonary arteries, respectively, to improve blood flow in addition to cardiac and respiratory function.

Two months later, the patient presented to a pediatric clinic with a swollen and red gastronomy tube site. On physical examination, the patient was febrile with a temperature of 39 °C. Intravenous ceftriaxone was administered while blood cultures were drawn. The patient was admitted to the Pediatric Cardiothoracic Intensive Care Unit and given intravenous clindamycin until the blood culture results returned negative for 48 h. Due to low bicarbonate levels, sodium citrate and citric acid were prescribed to address renal tubular acidosis. At 19 months of age, the patient presented to the emergency room (ER) with a 1-week history of cough, respiratory distress, and post-tussive emesis. The patient tested positive for influenza. Physical examination revealed mild tachypnea and coarse breath sounds bilaterally. Supportive care was provided, and the patient was discharged once oxygen saturation levels improved.

## Discussion

While this case cannot serve as the basis for generalized management recommendations, it can provide meaningful insight into rare and diagnostically complex conditions. This case illustrates how both advanced imaging and multidisciplinary care can be integrated to address the diverse anatomical and physiological challenges presented by isomerism of the right atrial appendages, a term now preferred over the historically used label of heterotaxy syndrome. The co-occurrence of isomerism of the right atrial appendages, total anomalous pulmonary venous return, and bilateral eparterial bronchi required individualized evaluation and decision-making to ensure optimal treatment. Historically grouped under the umbrella term “heterotaxy syndrome,” isomerism of the atrial appendages is now classified into right or left isomerism based on imaging findings [1, 2].

Congenital heart defects associated with isomerism of the atrial appendages are typically identified through fetal echocardiography, which allows for immediate medical and surgical intervention following birth [4]. Although fetal ultrasound is a useful tool for detecting isomerism, diagnosing the condition proves to be challenging. A study by Yim et al. found that > 20% of patients with isomerism of the atrial appendages presented with discordant findings between their atrial appendage arrangement, splenic status, and bronchopulmonary branching [4]. Such complex cases require independent evaluation of each organ system beyond fetal ultrasound to confirm a diagnosis of isomerism of the atrial appendages. In this case, diagnosis of isomerism of the right atrial appendages was supported by characteristic findings, including asplenia and bilateral tri-lobed lungs (Fig. 1).

In this case, a comprehensive assessment of the patient’s anatomy was performed through 3D reconstruction. Computed tomography angiography with 3D reconstruction identified congenital heart defects, such as atrioventricular septal defect, total anomalous pulmonary venous return, and discordant ventriculo-arterial connections with an anterior and right-sided aorta. Early recognition of asplenia was crucial, as it allowed for a timely initiation of prophylactic amoxicillin and prevented sepsis. Proper visualization of the patient’s heart through 3D reconstruction helped guide the placement of a Blalock-Taussig shunt as well as the following Glenn procedure to improve systemic and pulmonary circulation. While surgery improves outcomes for patients with isomerism of the right atrial appendages, considerations against surgical intervention of the pulmonary veins include pulmonary vein hypoplasia, elevated risk of postoperative pulmonary vein stenosis, and single ventricle physiology [3, 5]. The utility of computed tomography angiography in evaluating the patient’s anatomy and guiding surgical interventions for isomerism of the atrial appendages reflects how cardiac computed tomography permits rapid acquisition and high-quality visualization of the heart. Despite the advantages of cardiac computed tomography, the high dose of radiation required for imaging presents a significant disadvantage. Although cardiovascular magnetic resonance imaging avoids radiation and provides a detailed 3D view, it requires general anesthesia in younger patients and poses the risk of renal injury due to the use of gadolinium agents [6]. Recent studies have proposed high-pitch computed tomography as an alternative to standard cardiac computed tomography to reduce scanning times and radiation exposures, but minor losses in image quality may occur [7].

The presence of bilateral eparterial bronchi posed additional challenges when managing the patient’s health. Tracheobronchial branching anomalies have been shown to make airway management more difficult, especially during surgeries as anesthesia is administered [8]. Additionally, anatomical variations of the tracheobronchial branching have been associated with recurrent infections. This was observed in the patient’s return to the ER with low oxygen saturations, cough, and respiratory distress, which required supplemental oxygen and supportive care. Early detection of tracheobronchial anomalies is essential, and computed tomography is widely considered to be the gold-standard imaging modality due to its direct and non-invasive nature [8]. Previous studies have used bronchoscopy in conjunction with computed tomography imaging to identify tracheobronchial anomalies, suggesting this strategy may serve as a comprehensive approach [9].

Previous reviews have concluded that optimal care of children with isomerism of the atrial appendages requires a team of subspecialists to address complications, such as immunodeficiency, intestinal malrotation, respiratory ciliary impairment, and cardiovascular abnormalities [3]. This case highlights the role of multidisciplinary care in treating patients with isomerism of the right atrial appendages and bilateral eparterial bronchi, as collaboration among cardiologists, radiologists, surgeons, and nutritionists was crucial in addressing systemic complications along with cardiac and airway abnormalities. A meta-analysis by Marshall et al. recently found that children with circulatory defects, such as those found in isomerism of the atrial appendages, are more likely to have impaired relationships and experience anxiety and mood disorders [10]. The risk of psychological disorders underscores the need to expand multidisciplinary care for patients with isomerism of the right atrial appendages to include medical psychologists and mental health providers while using screening to detect behavioral symptoms.

Future research should continue to assess the causes and risk factors of isomerism of right atrial appendages. Although our case does not support generalized conclusions about the causes of isomerism of the right atrial appendages, previous studies have shown that genetic mutations, such as those affecting the Zic family member 3 gene involved in embryonic left–right axis patterning, and environmental factors, including maternal diabetes and teratogenic exposures, have been implicated in the development of isomerism of the atrial appendages [1]. Specifically, a recent study by Hagen et al. also identified genomic copy number variations across 56 genes in the nodal growth differentiation factor, bone morphogenic protein, and wingless-related integration site signaling pathways that were associated with isomerism of the atrial appendages, which reinforces the importance of body patterning pathways in establishing a proper left–right axis [1]. Hagen et al. demonstrated that genomic findings in patients with abnormal left–right patterning are only interpretable when patients are segregated by right vs. left atrial appendage isomerism [1]. Our patient had harmonious findings consistent with isomerism of the right atrial appendages, eliminating diagnostic uncertainty. When discordant findings occur, current multimodality imaging reliably allows accurate assessment and classification, an important consideration for medical providers. The patient exhibited right-handed ventricular topology, a critical feature in defining the overall segmental arrangement.

Additional research regarding the genetic and developmental factors associated with isomerism of the right or left atrial appendages will allow clinicians to recognize risk factors more effectively. This may potentially help with prevention, early diagnosis, and treatment strategies, ultimately improving long-term survival and quality of life for affected patients.

## Data Availability

No datasets were created or analyzed during the study.
